# Physicians’ Perspectives on Access to Innovative Oncology Therapies: A Cross-Sectional Survey in Bulgaria

**DOI:** 10.3390/healthcare14182926

**Published:** 2026-09-09

**Authors:** Iva Zdravkova-Aneva, Tihomir Dermendzhiev, Kostadin Kostadinov, Angel Prodanov, Rositsa Dimova, Ralitsa Raycheva

**Affiliations:** 1 Department of Social Medicine and Public Health, Medical University of Plovdiv, 4002 Plovdiv, Bulgaria; iva.zdravkova-aneva@mu-plovdiv.bg (I.Z.-A.); kostadinr.kostadinov@mu-plovdiv.bg (K.K.); 2 Department of Medical microbiology and immunology “Prof. Dr. Elissay Yanev”, Medical University of Plovdiv, 4002 Plovdiv, Bulgaria; drdermendzhiev@abv.bg; 3 Department of Anaesthesiology and Intensive Care Medicine, Medical University of Plovdiv, 4002 Plovdiv, Bulgaria; angel.prodanov@mu-plovdiv.bg; 4 Department of Health Management and Health Economics, Medical University of Plovdiv, 4002 Plovdiv, Bulgaria; rositsa.dimova@mu-plovdiv.bg

**Keywords:** innovative oncology therapies, patient access, health technology assessment, reimbursement, oncology, precision medicine, physicians, Regulation (EU) 2021/2282

## Abstract

**Highlights:**

**What are the main findings?**

- Bulgarian oncology physicians reported high self-reported professional readiness and broadly favorable perceptions of the clinical value of innovative oncology therapies.
- Professional experience and monthly patient volume were independently associated with more favorable perceptions of access. Financial, regulatory, HTA-related, and organizational factors were the most frequently reported perceived barriers.

**What are the implications of the main findings?**

- Respondents identified reimbursement processes, HTA implementation, molecular diagnostic capacity, clinical-research opportunities, and access to specialized oncology services as important areas for improvement.
- The findings may inform policy discussions but represent physicians’ perceptions rather than objective measurements or causal determinants of access.

**Abstract:**

Background/Objectives: Despite major advances in precision medicine, equitable and timely access to innovative oncology therapies remains challenging because of differences in reimbursement systems, health technology assessment (HTA), and healthcare organization. Physicians are well positioned to identify barriers affecting the implementation of these therapies in routine practice. This study evaluated Bulgarian physicians’ perceptions of innovative oncology therapies, assessed patient access, identified barriers to timely availability, and examined factors perceived as associated with these perceptions. Methods: A cross-sectional web-based survey was conducted between March and May 2025 among physicians involved in oncology care in Bulgaria. The questionnaire comprised six domains addressing professional characteristics, self-perceived knowledge, clinical implementation, perceptions of patient access, barriers to implementation, and policy priorities. Descriptive statistics, Pearson’s chi-square tests, Cramér’s V, and multivariable ordinal logistic regression were used to analyze the data. Results: A total of 114 physicians participated. Respondents generally reported moderate-to-high levels of self-perceived knowledge regarding innovative oncology therapies. Self-perceived knowledge was higher among medical oncologists (adjusted *p* < 0.001) and physicians treating at least 100 patients with cancer per month (adjusted *p* = 0.003). Overall, 57.9% of respondents (95% CI: 48.7–66.6%) rated the perceived timeliness of access as good or very good, while 68.4% (95% CI: 59.4–76.2%) reported encountering patients who sought treatment abroad because innovative therapies were perceived as unavailable or insufficiently accessible in Bulgaria. Professional experience (aOR = 0.68, 95% CI: 0.50–0.93; *p* = 0.014) and higher monthly patient volume (aOR = 0.45, 95% CI: 0.21–0.96; *p* = 0.039) were independently associated with more favorable perceptions of access. Financial constraints, regulatory and HTA-related delays, and perceived delays in pharmaceutical-company submissions were the principal perceived barriers. Conclusions: Bulgarian oncology specialists demonstrate high self-reported professional readiness for innovative oncology therapies; however, the perceived timely patient access remains constrained primarily by financial, organizational, and reimbursement-related factors. Although, these findings reflect physicians’ perceptions and do not establish causal determinants of access, strengthening reimbursement pathways, HTA implementation, molecular diagnostic capacity, and clinical research infrastructure may improve equitable access to innovative oncology therapies within the evolving European HTA framework.

## 1. Introduction

Cancer remains one of the leading causes of morbidity and mortality worldwide, accounting for a substantial clinical, social, and economic burden. According to the World Health Organization, cancer is responsible for nearly one in six deaths globally, and its incidence continues to increase because of population aging, demographic changes, and improvements in diagnosis [1,2]. Advances in molecular biology, immunology, and precision medicine have transformed oncology over the past two decades, resulting in an unprecedented number of innovative medicinal products entering clinical practice. These include immune checkpoint inhibitors, targeted therapies, antibody-drug conjugates, cell and gene therapies, and biomarker-driven treatment strategies that have substantially improved survival and quality of life for many patients [3,4,5,6,7].

Despite these advances, equitable and timely access to innovative oncology therapies remains a major challenge worldwide. Although the European Medicines Agency (EMA) provides centralized marketing authorization for innovative medicinal products, patient access varies considerably across European countries. Differences in health technology assessment (HTA) procedures, pricing and reimbursement policies, healthcare expenditure, diagnostic capacity, and organizational readiness contribute to substantial inequalities in the availability and uptake of innovative cancer medicines [8,9,10,11].

Recognizing these challenges, the European Union (EU) adopted Regulation (EU) 2021/2282 on Health Technology Assessment, which introduced Joint Clinical Assessments to improve cooperation among Member States, reduce duplication of assessments, and facilitate more efficient patient access to innovative health technologies [12]. Nevertheless, reimbursement decisions remain the responsibility of individual Member States [12,13], and important differences in access continue to exist because of variations in healthcare financing, budgetary capacity, infrastructure, and national healthcare priorities [9,14].

In Bulgaria, access to innovative oncology therapies is influenced by multiple interrelated factors, including lengthy reimbursement procedures, limited public healthcare resources, budget impact considerations, restricted availability of molecular diagnostics, and organizational barriers within the healthcare system [15,16,17,18]. The implementation of precision oncology further requires timely access to biomarker testing, molecular diagnostics, multidisciplinary expertise, and specialized oncology centers, all of which remain unevenly available across healthcare systems [11,19].

Physicians play a pivotal role in the successful implementation of innovative oncology therapies. As the primary decision-makers responsible for treatment selection, monitoring, and patient management, oncologists and other oncology specialists are uniquely positioned to identify practical barriers that may not be evident from administrative or reimbursement data alone. Their perceptions therefore represent an important but underutilized source of evidence for healthcare policy, reflecting challenges related to reimbursement procedures, diagnostic infrastructure, multidisciplinary collaboration, patient education, and organizational capacity.

Although several international studies have investigated clinicians’ perspectives on precision oncology, genomic medicine, and molecular diagnostics [20,21,22], relatively little evidence is available regarding physicians’ perceptions of overall access to innovative oncology therapies, particularly in Central and Eastern Europe. Bulgaria provides an informative setting because of its centralized reimbursement process, universal health insurance system, and ongoing implementation of the new European HTA Regulation. Understanding physicians’ perspectives within this context may therefore provide valuable evidence for countries facing similar healthcare challenges.

We hypothesized that physicians would perceive organizational and financing barriers as more influential determinants of patient access than physician-related factors.

The aim of this study was to evaluate physicians’ perceptions regarding innovative oncology therapies in Bulgaria, assess their views on the current level of patient access, identify the major barriers perceived as limiting timely availability of innovative medicines, and examine whether these perceptions differ according to demographic and professional characteristics. By providing evidence from a cross-sectional survey, this study seeks to contribute to the international literature on access to innovative oncology therapies and to support evidence-informed policy initiatives aimed at improving equitable access to cancer innovation.

## 2. Materials and Methods

### 2.1. Study Design and Setting

A cross-sectional survey was conducted to investigate physicians’ perceptions regarding innovative oncology therapies, patient access, barriers to implementation, and priorities for improving access within the Bulgarian healthcare system. The study was designed as an anonymous, questionnaire-based survey and followed the principles of the Strengthening the Reporting of Observational Studies in Epidemiology (STROBE) statement for reporting observational studies.

Data collection was performed between March and May 2025 using a secure web-based questionnaire developed specifically for the purposes of this study. The survey targeted physicians involved in the diagnosis, treatment, and follow-up of patients with malignant diseases across Bulgaria. A convenience sampling strategy was used.

### 2.2. Participants and Recruitment

Eligible participants were physicians actively involved in oncology care, including medical oncologists, clinical hematologists, radiation oncologists, surgeons, nuclear medicine specialists, and other physicians participating in multidisciplinary cancer management. Physicians were recruited through national professional organizations, scientific societies, oncology departments, and professional mailing lists. Recruitment sought participation from physicians working in different oncology-related specialties and healthcare sectors across Bulgaria. However, convenience sampling was used, and the sampling frame was not designed to generate a nationally representative sample.

Invitations containing information about the study objectives and an electronic survey link were distributed electronically. Participation was voluntary, anonymous, and without financial incentives. Before accessing the questionnaire, all participants received written information regarding the study and were required to provide informed electronic consent. Responses from participants who did not provide consent were excluded from the analysis.

### 2.3. Survey Instrument

The questionnaire was developed following a comprehensive review of the scientific literature on innovative oncology therapies, health technology assessment (HTA), market access, precision oncology, and physicians’ perceptions of oncology innovation. Existing international surveys and published evidence were considered during questionnaire development. The literature review was used to inform item selection and domain coverage.

The final questionnaire consisted of six thematic domains:

1. Demographic and professional characteristics;
2. Self-perceived knowledge and sources of information regarding innovative oncology therapies;
3. Clinical implementation and perceived clinical value;
4. Perceptions of patient access to innovative oncology therapies in Bulgaria;
5. Barriers perceived as limiting timely patient access;
6. Policy recommendations for improving access.

Most attitudinal questions were measured using five-point Likert scales ranging from very low to very high importance or from strong disagreement to strong agreement, depending on the specific construct being assessed. Several questions allowed multiple responses where appropriate. Before answering the relevant questionnaire items, all participants were given an explicit definition of “innovative oncology therapy.” For the purposes of the survey, the term was defined as a medicinal product used in oncology that contains an active substance or a combination of active substances that had not previously undergone an authorization procedure by the European Medicines Agency. The definition was displayed immediately before the questions concerning innovative oncology therapies, together with a link to the corresponding EMA glossary entry. For the purposes of this study, self-reported professional readiness was defined as physicians’ perceived level of being informed about innovative oncology therapies and their perceived preparedness to engage with these therapies in clinical practice. It was assessed through self-report questionnaire items and did not constitute an objective evaluation of knowledge, clinical competence, or professional performance.

No pilot testing, cognitive interviewing, formal external expert-panel review, or formal psychometric validation was performed. Cronbach’s alpha was not calculated because the questionnaire was not designed as a unidimensional psychometric scale and no composite domain scores were constructed. The complete questionnaire, including all items and response options, is provided as Supplementary Material.

### 2.4. Variables

The primary outcomes of interest were physicians’ perceptions regarding:

- Self-reported professional readiness for innovative oncology therapies;
- Perceived patient access to innovative oncology therapies;
- Barriers perceived as limiting timely patient access;
- Policy measures considered most important for improving access.

Independent variables included physician sex, age, professional experience, medical specialty, healthcare sector, monthly oncology patient volume, patient population, and proportion of professional activity devoted to clinical care.

For inferential analyses, several variables were categorized to facilitate statistical comparisons. Professional experience was grouped into predefined categories, while monthly patient volume was dichotomized into physicians treating fewer than 100 versus at least 100 oncology patients per month. Responses recorded on five-point Likert scales were collapsed into three categories (low, moderate, and high) where appropriate to satisfy the assumptions of contingency table analyses.

Because respondents could report more than one medical specialty, the complete descriptive specialty profile was analyzed as a multiple-response variable, and the corresponding percentages could exceed 100%. For inferential analyses, specialty was recoded into two mutually exclusive categories. Respondents who selected medical oncology, either alone or together with another specialty, were classified as medical oncologists; all respondents who did not select medical oncology were classified as other oncology-related specialists.

### 2.5. Statistical Analysis

Categorical variables were summarized using frequencies and percentages, whereas continuous variables were described using medians and interquartile ranges (IQR) because several variables demonstrated non-normal distributions. Principal descriptive proportions are presented with two-sided 95% confidence intervals calculated using the Wilson score method. Confidence intervals were calculated using the number of respondents with available data for the corresponding variable or questionnaire item; for subgroup analyses, the relevant subgroup sample size was used as the denominator. The continuous variables were described using medians and interquartile ranges (IQR) because several variables demonstrated non-normal distributions.

Associations between categorical variables were evaluated using Pearson’s chi-square (χ^2^) test. Effect sizes were quantified using Cramér’s V and interpreted according to conventional criteria, with values of approximately 0.10, 0.30, and 0.50 representing small, moderate, and large associations, respectively. Differences in ordinal outcomes across physician characteristics were initially explored using contingency-table analyses.

Subsequently, multivariable ordinal logistic regression was performed to identify independent predictors of physicians’ perceptions regarding the timeliness of patient access to innovative oncology therapies. Predictor variables included physician sex, professional experience, medical specialty, healthcare sector, and monthly oncology patient volume. Age was not included in the model because of its close conceptual and empirical relationship with professional experience. Formal multicollinearity diagnostics were not performed for the remaining predictors. Adjusted odds ratios (aORs) with 95% confidence intervals (CI) were calculated. The proportional-odds assumption was evaluated using the test of parallel lines; a non-significant result was interpreted as indicating that the effects of the predictors were consistent across the cumulative outcome thresholds.

Missing values were not imputed. Descriptive analyses were based on the available observations for each variable, whereas inferential analyses were performed using complete cases for the variables included in the respective analysis. Responses omitted because of questionnaire skip patterns were treated as not applicable rather than as missing data.

The Benjamini–Hochberg procedure was applied to the exploratory bivariate comparisons to control the false discovery rate at 5%. The corresponding adjusted *p*-values are reported for these analyses. The *p*-values from the prespecified multivariable ordinal logistic regression model were not included in this adjustment. All statistical tests were two-sided, and an adjusted *p*-value < 0.05 was considered statistically significant for the exploratory bivariate comparisons. Statistical analyses were performed using IBM SPSS Statistics version 26 (IBM Corp., Armonk, NY, USA).

## 3. Results

### 3.1. Characteristics of the Study Population

Of the 247 physicians invited to participate, 115 completed the online questionnaire, yielding a response rate of 46.6%. One respondent did not provide informed consent and was excluded, resulting in a final analytical sample of 114 physicians. Four respondents (3.5%) had partial demographic data. Age was missing for two respondents and professional experience for three, with one respondent missing both variables. Age was therefore available for 112 participants and professional experience for 111. No missing values were imputed, and descriptive analyses were based on the available observations for each variable.

The study population comprised equal proportions of men and women (50.0% each; 95% CI: 41.0–59.0%). The median age was 45 years (IQR, 34–57 years), while the median professional experience in oncology was 15 years (IQR, 7.5–25 years). Among the 111 respondents with available professional-experience data, 28.8% (95% CI: 21.2–37.9%) had between 11 and 20 years of oncology practice, whereas 13.5% (95% CI: 8.4–21.1%) reported more than 30 years of professional experience.

Medical oncologists represented the largest professional group (44.7%; 95% CI: 35.9–53.9%), followed by surgeons (23.7%; 95% CI: 16.8–32.3%), radiation oncologists (10.5%; 95% CI: 6.1–17.5%), clinical hematologists (7.0%; 95% CI: 3.6–13.2%), and nuclear medicine specialists (6.1%; 95% CI: 3.0–12.1%). Because respondents could report multiple specialties, the sum of the percentages for the complete specialty profile could exceed 100%.

Most physicians treated patients with solid tumors (94.7%; 95% CI: 89.0–97.6%) and adult patients (96.5%; 95% CI: 91.3–98.6%). Nearly two-thirds worked predominantly in the public healthcare sector (60.5%; 95% CI: 51.4–69.0%), while approximately one-third practiced mainly in private institutions (35.1%; 95% CI: 26.9–44.2%). Clinical activity was substantial, with 64.9% (95% CI: 55.8–73.1%) dedicating at least 80% of their working time to patient care and 46.5% (95% CI: 37.6–55.6%) managing at least 100 patients with cancer per month (Table 1).

### 3.2. Self-Perceived Knowledge of Innovative Oncology Therapies and Trusted Information Sources

Most respondents reported moderate-to-high self-rated levels of knowledge regarding available innovative oncology therapies. Scientific conferences and seminars were the most frequently used sources of information, followed by specialized medical literature, pharmaceutical companies, online resources and databases, national and international oncology organizations, educational courses, and professional expert networks.

Although scientific conferences and seminars represented the most frequently used source of information, specialized medical literature was perceived as the most credible source, with 89.5% of respondents (95% CI: 82.5–93.9%) rating it as highly or very highly credible. Scientific conferences and seminars received similarly positive ratings from 84.2% of respondents (95% CI: 76.4–89.8%). National and international oncology organizations were rated as highly or very highly credible by 73.7% (95% CI: 64.9–80.9%), followed by educational courses and workshops by 68.4% (95% CI: 59.4–76.2%), professional and expert networks by 66.7% (95% CI: 57.6–74.7%), online resources and databases by 64.0% (95% CI: 54.9–72.3%), and pharmaceutical companies by 62.3% (95% CI: 53.1–70.6%).

Self-perceived knowledge was significantly higher among medical oncologists than among physicians from other oncology-related specialties (χ^2^(3) = 23.59, *p* < 0.001, Cramér’s V = 0.455). Among medical oncologists, 58.8% (95% CI: 45.2–71.2%) considered themselves fully informed, compared with 17.5% (95% CI: 10.0–28.6%) of other oncology-related specialists.

Clinical workload was also associated with self-perceived knowledge. Physicians treating at least 100 patients with cancer per month were more likely to consider themselves fully informed than those treating fewer than 100 patients per month (χ^2^(3) = 14.25, *p* = 0.003, Cramér’s V = 0.353). Among physicians with the higher patient volume, 52.8% (95% CI: 39.7–65.6%) considered themselves fully informed, compared with 21.3% (95% CI: 12.9–33.1%) of those with the lower patient volume. Conversely, the response “informed to some extent” was more frequent among respondents treating fewer than 100 patients per month.

Professional experience was not significantly associated with overall self-rated knowledge. It was, however, associated with the perceived credibility of online resources and databases, χ^2^(4) = 10.76, *p* = 0.029, Cramér’s V = 0.220. Specialists with less than 10 years of professional experience more frequently rated these sources as moderately credible than those with 10–24 years of experience. The magnitude of this association was small to moderate. No significant associations were identified between overall self-perceived knowledge and sex, healthcare sector, the age profile of treated patients, or the proportion of working time devoted to clinical activities.

### 3.3. Clinical Implementation and Perceived Value of Innovative Oncology Therapies

Innovative oncology therapies were routinely incorporated into clinical practice, although the proportion of patients receiving such treatments varied among respondents. Improvements in survival, quality of life, opportunities for personalized treatment, and the possibility of combination therapy were among the most frequently identified benefits of innovative therapies.

Clinical value was assessed using time-to-event outcomes, measures of therapeutic response, and patient-reported outcomes. Overall survival and progression-free survival received the strongest ratings among the time-to-event outcomes. Among measures of therapeutic response, objective response, overall response, and disease control were consistently considered important. Patient-reported improvements in quality of life and symptom reduction were also highly valued.

The assessments of clinical value demonstrated substantial consistency across professional groups. Professional experience was not significantly associated with the ratings assigned to overall survival, progression-free survival, event-free survival, disease-free survival, one-year survival, five-year survival, time to progression, time to response, duration of response, or time to subsequent therapy. Similarly, no significant associations with professional experience were identified for objective response, overall response, pathological complete response, disease control, minimal residual disease, recurrence, symptom reduction, improvement in quality of life, functional improvement, or reduction in adverse effects.

Although respondents acknowledged the relevance of pharmacoeconomic evidence, clinical benefit remained the dominant dimension in their assessment of innovative therapies. These findings demonstrate broad professional consensus regarding the clinical value of oncology innovation, irrespective of years of experience.

### 3.4. Perceived Access to Innovative Oncology Therapies

Perceptions of access demonstrated greater variability than assessments of clinical value. Overall, 57.9% of respondents (95% CI: 48.7–66.6%) rated the timeliness of introducing innovative oncology therapies in Bulgaria as very good or good, 28.9% (95% CI: 21.4–37.9%) rated it as satisfactory, and 13.2% (95% CI: 8.1–20.6%) rated it as poor or very poor.

When comparing access in Bulgaria with that in other European countries, 31.6% of respondents (95% CI: 23.8–40.6%) considered access faster or much faster, 36.0% (95% CI: 27.7–45.1%) considered it comparable, and 32.5% (95% CI: 24.6–41.5%) considered it slower or much slower.

Limitations in access were also apparent in clinical practice. More than two-thirds of respondents reported encountering patients who sought treatment abroad because an innovative therapy was unavailable or insufficiently accessible in Bulgaria.

In the multivariable ordinal logistic regression model, professional experience and monthly patient volume were independently associated with perceived timeliness of access (Table 2). Each additional 10 years of professional experience was associated with a 32% reduction in the odds of reporting a less favorable assessment of timeliness (aOR = 0.68, 95% CI 0.50–0.93; *p* = 0.014). Specialists treating at least 100 patients with cancer per month also had lower odds of providing an unfavorable assessment than those treating fewer than 100 patients per month (aOR = 0.45, 95% CI 0.21–0.96; *p* = 0.039). Medical oncologists tended to evaluate access more favorably than other oncology-related specialists, although this association did not reach statistical significance (aOR = 0.50, 95% CI 0.23–1.10; *p* = 0.086). Sex and healthcare sector were not independently associated with perceived timeliness. The overall model was statistically significant, χ^2^(5) = 16.29, *p* = 0.006, with a Nagelkerke pseudo-R^2^ of 0.146. The multivariable ordinal logistic regression included 111 respondents with complete data for the outcome and all five predictors. The test of parallel lines was not statistically significant (χ^2^(15) = 10.43, *p* = 0.792), indicating no evidence that the proportional-odds assumption was violated.

### 3.5. Perceived Barriers to Timely Access

Respondents identified system-level factors perceived as the major barriers to timely access to innovative oncology therapies.

The most frequently selected perceived barrier was the high budget impact on the National Health Insurance Fund, reported by 57.0% of respondents (95% CI: 47.8–65.7%). Regulatory and health technology assessment delays were reported by 48.2% (95% CI: 39.3–57.3%), while perceived delays in pharmaceutical-company submissions for inclusion in the Positive Drug List were identified by 29.8% (95% CI: 22.2–38.8%). Insufficient information and training of medical staff were selected by 20.2% (95% CI: 13.8–28.5%), and limited patient awareness and understanding by 17.5% (95% CI: 11.7–25.6%).

Ratings of the perceived importance of these barriers demonstrated a similar hierarchy. High budget impact received the strongest assessments, with 62.3% of respondents (95% CI: 53.1–70.6%) rating it as highly or very highly important. Financial and regulatory barriers were consequently perceived as more important than educational barriers.

Professional experience was significantly associated with the perceived importance of insufficient information and training among medical staff, χ^2^(4) = 12.98, *p* = 0.011, Cramér’s V = 0.242. Specialists with 10–24 years of experience more frequently rated this barrier as highly important than respondents with at least 25 years of experience. The effect size indicated a small-to-moderate association.

Professional experience was also associated with the perceived importance of insufficient patient awareness and understanding, χ^2^(4) = 11.89, *p* = 0.018, Cramér’s V = 0.231. Respondents with at least 25 years of experience more frequently rated this barrier as moderately important than those with 10–24 years of experience.

No significant differences according to professional experience were observed in the perceived importance of pharmaceutical company submission delays or regulatory and HTA perceived delays. A borderline association was identified for the high budget impact on the National Health Insurance Fund, but no clearly distinguishable differences between individual experience groups were demonstrated.

### 3.6. Policy Recommendations for Improving Access

Respondents expressed strong support for measures intended to improve access to innovative oncology therapies. Expanding opportunities for participation in clinical trials was rated as highly or very highly important by 56.1% of respondents (95% CI: 47.0–64.9%), while 30.7% (95% CI: 23.0–39.7%) rated this measure as moderately important. Improving access to specialized oncology centers was rated as highly or very highly important by 60.5% of respondents (95% CI: 51.4–69.0%), while a further 28.9% (95% CI: 21.4–37.9%) rated it as moderately important.

Approximately two-thirds of respondents supported the creation of a dedicated fund for the reimbursement of innovative oncology therapies, although many emphasized that such a fund should operate according to clearly defined eligibility, financing, and governance criteria.

Professional experience was significantly associated with the perceived importance of conducting more clinical trials locally, χ^2^(4) = 9.54, *p* = 0.049, Cramér’s V = 0.207. Specialists with 10–24 years of experience more frequently assigned low importance to this measure than those with less than 10 years of experience. The effect size was small.

Professional experience was not significantly associated with the perceived importance of improving access to specialized oncology centers or with support for establishing a dedicated reimbursement fund. Most policy measures therefore received broadly consistent support across professional groups.

Taken together, the respondents favored structural reforms targeting financing, reimbursement procedures, clinical research capacity, and specialized oncology infrastructure as the predominant strategies for improving equitable and timely access to innovative therapies.

**Figure 1:**
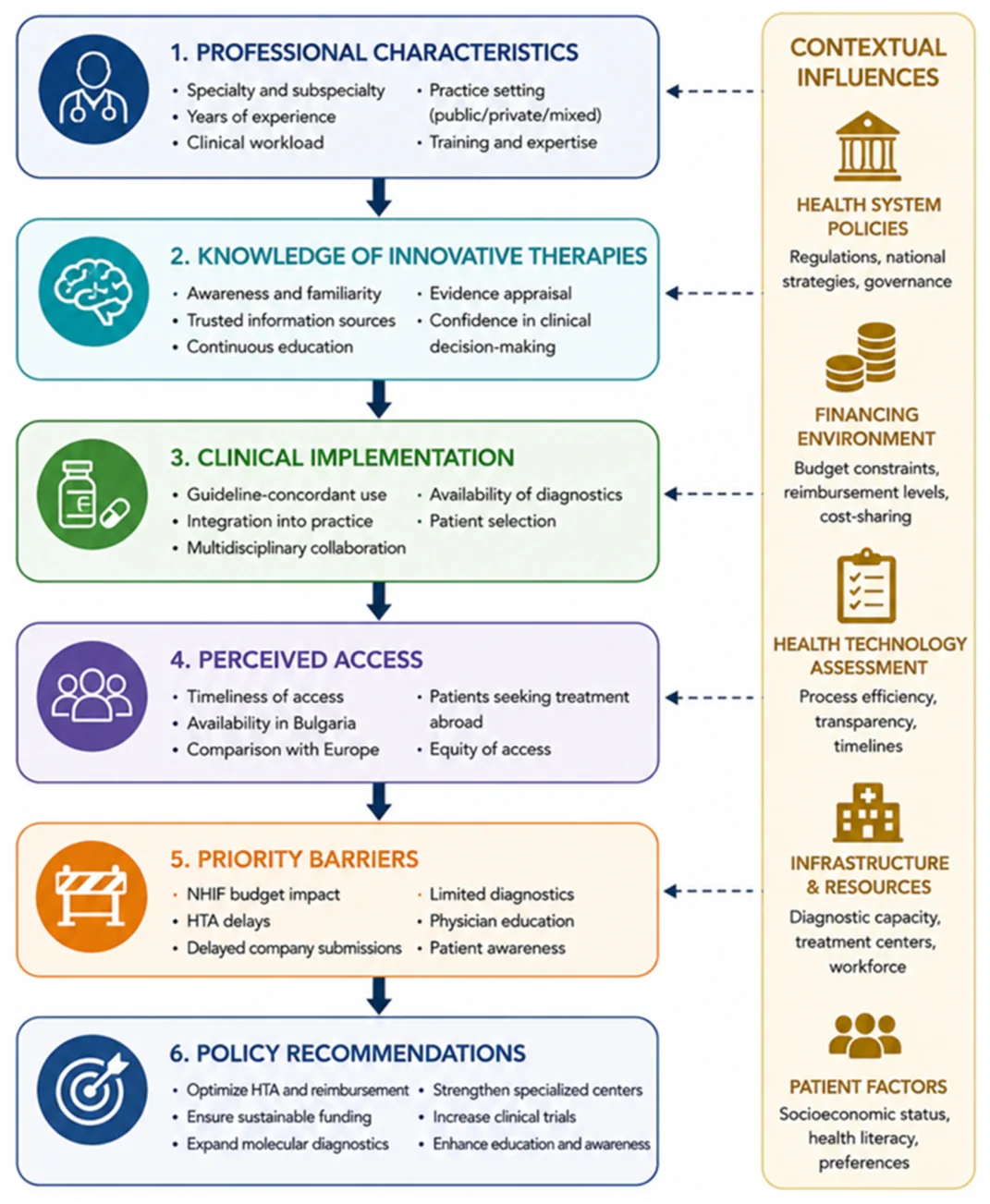
Proposed framework of factors perceived as influencing access to innovative oncology therapies based on the findings of the present study.

The relationships among physician characteristics, self-perceived knowledge, access, barriers, and proposed policy interventions are summarized in Figure 1.

## 4. Discussion

### 4.1. Key Findings

This multidisciplinary survey examined Bulgarian oncology specialists’ perceptions of innovative therapies, including self-reported professional readiness, clinical implementation, patient access, perceived barriers, and priorities for health-system improvement.

Four main findings emerged. First, respondents generally reported high professional readiness, particularly medical oncologists and physicians managing larger numbers of patients with cancer. Second, despite recognizing the clinical value of innovative therapies, respondents expressed less favorable views of patient access. Third, financial and organizational constraints—including anticipated budget impact on the National Health Insurance Fund, perceived HTA and reimbursement delays, and delays in pharmaceutical-company submissions—were the most frequently reported barriers. Finally, respondents supported improvements in reimbursement, clinical research, molecular diagnostics, and specialized oncology services.

These findings represent physicians’ perceptions rather than objective measurements of access. Nevertheless, they suggest that participating clinicians perceived health-system organization, financing, and market-access processes as more important barriers than physician-related factors.

### 4.2. Self-Reported Professional Readiness for Oncology Innovation

Respondents generally reported high self-perceived knowledge and professional readiness regarding innovative oncology therapies. Medical oncologists and physicians managing higher monthly patient volumes reported greater self-perceived knowledge than respondents from other specialties and those with lower patient volumes. Because the survey did not objectively assess knowledge, competence, exposure to innovative treatments, or the mechanisms underlying these associations, the findings should be interpreted as differences in self-assessment rather than demonstrated differences in professional readiness.

Previous research has similarly associated clinical experience and disease-specific expertise with guideline adherence, use of precision-medicine approaches, and patient outcomes [20,23,24]. Participation in multidisciplinary care, continuing education, and engagement with scientific literature may also support clinicians’ familiarity with emerging evidence [25,26,27]. However, the present study did not objectively assess knowledge or competence.

Scientific literature, clinical guidelines, professional organizations, and scientific meetings were regarded as the most credible information sources. Similar preferences have been reported among oncologists internationally, despite the increasing availability of digital educational resources [27,28]. ESMO has also emphasized continuous professional development as an important requirement for the safe implementation of increasingly complex oncology treatments [29].

Professional experience was associated only with the perceived credibility of online resources, with younger physicians assigning greater value to digital platforms. This finding is consistent with reported generational differences in physicians’ information-seeking behavior [30,31,32]. Digital resources are becoming important components of lifelong learning, provided that they are evidence-based and integrated with established clinical guidance [32,33].

Respondents also showed broad agreement regarding the clinical outcomes that should guide assessments of innovative therapies, prioritizing survival, disease control, quality of life, and patient-reported outcomes. This is consistent with contemporary oncology value frameworks that combine survival endpoints with patient-centered outcomes [34] and with the increasing use of patient-reported outcomes in regulatory and HTA decision-making [35,36].

Overall, the findings indicate relatively high self-reported readiness among participating physicians. Differences in self-perceived knowledge were associated mainly with specialty and clinical workload, but they should not be interpreted as objectively demonstrated differences in knowledge or competence.

### 4.3. Access to Innovative Oncology Therapies in the Bulgarian and European Context

In contrast to their generally favorable self-assessments of professional readiness, respondents expressed more variable views of patient access. Although more than half rated the timeliness of introducing innovative therapies as good or very good, approximately one-third perceived access in Bulgaria as slower than in other European countries. More than two-thirds reported encountering patients who sought treatment abroad because innovative therapies were perceived as unavailable or insufficiently accessible in Bulgaria. These reports were not independently verified.

International evidence provides context for these perceptions. Access to newly authorized oncology medicines varies considerably across Europe, with Central and Eastern European countries often experiencing longer reimbursement timelines than Western European countries [37,38,39,40]. The EFPIA Patients W.A.I.T. Indicator has similarly documented cross-country differences in the availability of innovative medicines, particularly in healthcare systems with more limited resources and lower pharmaceutical expenditure [41,42].

Respondents’ reports of patients seeking treatment abroad are also consistent with literature describing cross-border access to unavailable treatments, clinical trials, compassionate-use programs, and named-patient mechanisms [43,44,45,46]. However, the present study did not collect patient-level data and cannot determine the frequency, reasons, or outcomes of cross-border treatment.

In the multivariable analysis, greater professional experience and higher monthly patient volume were associated with more favorable perceptions of access. The study did not assess clinicians’ familiarity with reimbursement procedures, use of alternative access pathways, participation in referral or research networks, or access to multidisciplinary services; therefore, the mechanisms underlying these associations remain uncertain. Medical oncologists also tended to report more favorable perceptions, but this association was not statistically significant after adjustment and should be interpreted cautiously. Previous studies have described differences among oncology specialties in attitudes toward therapeutic innovation and healthcare-resource allocation [47,48], but the present study cannot determine whether the same perceived factors explain the observed pattern.

These findings also illustrate the distinction between regulatory authorization and effective access. EMA authorization is followed by HTA, price negotiation, reimbursement, and national implementation. Analyses of the European HTA Regulation indicate that joint clinical assessments alone are unlikely to eliminate access inequalities without improvements in national financing, reimbursement, and implementation processes [14,49].

Thus, respondents perceived a gap between professional readiness and timely access. Objective studies of reimbursement timelines, hospital-level availability, diagnostic capacity, and patient access are needed to determine whether these perceptions correspond to measurable delays.

### 4.4. Priority Barriers Perceived as Limiting Timely Access to Innovative Oncology Therapies

Respondents most frequently identified systemic rather than clinical barriers. Anticipated budget impact on the NHIF, perceived HTA and reimbursement delays, and delays in pharmaceutical-company submissions were rated as more important than physician education or patient awareness. These findings reflect clinicians’ perceptions and should not be interpreted as direct measurements of the magnitude or duration of these barriers.

The prominence of financial and reimbursement-related concerns is consistent with international evidence showing that reimbursement policies, pricing mechanisms, and healthcare resources contribute to inequalities in access to innovative oncology medicines [8,14,50]. Respondents’ emphasis on HTA and reimbursement is also relevant to Regulation (EU) 2021/2282. Although joint clinical assessments may reduce duplication, pricing, reimbursement, budget allocation, and implementation remain national responsibilities [12,49,51].

Physician education was considered less influential than financing and organizational constraints, although respondents continued to recognize the importance of professional development. International evidence similarly indicates that education and guideline adherence remain important but cannot compensate for reimbursement delays or insufficient healthcare resources [8,52,53].

Professional experience influenced perceptions of physician education and patient awareness, but the corresponding effect sizes were relatively small. These findings suggest broad agreement across professional groups regarding the relative importance of systemic barriers.

More generally, clinical innovation produces patient benefit only when healthcare systems can incorporate it into routine practice. Countries with similar regulatory standards may still experience different levels of access because of variation in HTA implementation, financing, procurement, and organizational capacity [9,54,55,56]. The respondents’ perceptions therefore reflect challenges that have also been described in other European healthcare systems.

### 4.5. Policy Implications

Respondents supported measures aimed at improving reimbursement procedures, expanding specialized oncology services, strengthening molecular diagnostic capacity, and increasing participation in clinical trials. These priorities should be understood as clinicians’ recommendations rather than evidence that the proposed measures will necessarily improve access.

Support for clinical research is particularly relevant because clinical trials can provide access to therapies before routine reimbursement while strengthening national research capacity and multidisciplinary collaboration. Limited access to oncology trials has been associated with inequalities in cancer care, particularly in Central and Eastern Europe [9,57,58,59].

Respondents also emphasized molecular diagnostics and specialized oncology services. Timely genomic and biomarker testing is increasingly important for selecting targeted treatments, and disparities in diagnostic infrastructure may limit the implementation of precision oncology even when medicines are available [11,60,61].

The findings are also relevant to the implementation of the European HTA framework. Joint clinical assessments may improve the efficiency of evidence evaluation, but their effect on patient access will depend on national reimbursement, financing, diagnostic, and implementation capacity. Coordination among regulatory authorities, HTA agencies, payers, clinicians, and patient organizations will therefore remain important [49,55].

Overall, participating physicians favored an integrated approach combining reimbursement reform, HTA implementation, diagnostic infrastructure, specialized services, and clinical-research capacity. These priorities may inform policy discussions, but their effects should be evaluated using objective indicators of reimbursement timelines, treatment availability, healthcare expenditure, diagnostic access, and patient outcomes.

Because the study did not evaluate the mechanisms through which the proposed policy measures might affect access, these recommendations should be regarded as priorities reported by participating physicians rather than evidence of intervention effectiveness.

### 4.6. Strengths and Limitations

To our knowledge, this is the first multidisciplinary survey conducted across Bulgaria to evaluate physicians’ perceptions of innovative oncology therapies, patient access, barriers, and policy priorities within the Bulgarian healthcare system. By including respondents from multiple oncology-related disciplines and healthcare sectors, the study captures a range of perspectives among clinicians involved in cancer care. However, because convenience sampling was used, the sample should not be regarded as nationally representative. The use of inferential statistical analyses, including multivariable ordinal logistic regression, allowed the identification of factors independently associated with physicians’ perceptions, extending the analysis beyond purely descriptive reporting.

An additional strength is the policy relevance of the study. The survey was conducted during a period of substantial transformation of the European HTA landscape following the implementation of Regulation (EU) 2021/2282. Consequently, the findings provide timely evidence regarding clinicians’ perspectives on access to innovative oncology therapies and may contribute to ongoing discussions concerning reimbursement pathways, joint clinical assessments, and oncology services in Bulgaria.

Several limitations should be acknowledged. First, the cross-sectional design captured physicians’ perceptions at a single point in time and did not permit conclusions regarding temporal changes or causal relationships. Because innovative therapies, reimbursement policies, and HTA procedures continue to evolve, physicians’ opinions may change in response to developments in the healthcare environment.

Second, the study relied on self-reported data. Although physicians are well positioned to describe their experiences with the implementation of innovative oncology therapies in routine practice, their responses may have been influenced by subjective perceptions, professional experience, or recall bias. The survey therefore reflects clinicians’ assessments rather than objective measures of self-rated level of knowledge, professional competence, medicine availability, reimbursement timelines, or patient access.

Third, the questionnaire was developed specifically for this study and did not undergo pilot testing, cognitive interviewing, or formal psychometric validation. Consequently, potential limitations related to item wording, interpretation, and measurement properties cannot be excluded. The questionnaire comprised conceptually heterogeneous items that were analyzed individually rather than combined into composite scales; therefore, assessment of internal consistency using Cronbach’s alpha was not applicable.

Fourth, the study used convenience sampling and was not designed to produce a nationally representative sample of physicians involved in oncology care in Bulgaria. Although respondents represented multiple oncology-related specialties and both public and private healthcare sectors, comprehensive national data permitting comparison of the sample with the target population by specialty, healthcare sector, geographical region, institutional type, and oncology-center volume were not available. Participation was voluntary, and physicians with a greater interest in innovative oncology therapies or health policy may have been more likely to participate. Selection bias, disproportionate participation by particular professional groups or institutions, and limited generalizability to the wider population of Bulgarian physicians involved in oncology care therefore cannot be excluded.

Finally, the study was conducted within the Bulgarian healthcare system. Although several of the perceived barriers—including perceived reimbursement delays, budget constraints, and organizational challenges—are consistent with observations reported in other European countries, the results should be interpreted within the Bulgarian regulatory, financing, and organizational context. Generalization to other healthcare systems should therefore be undertaken with caution.

In the future, larger study groups must be examined to validate the findings reported here; prospective sample-size estimates indicate that at least 134 complete observations would be required to achieve 80% power at an alpha level of 0.05 under the specified assumptions.

Despite these limitations, the study captures multidisciplinary clinicians’ perspectives on access to innovative oncology therapies in Bulgaria and identifies areas that warrant further investigation using representative samples, validated instruments, and objective indicators of reimbursement, treatment availability, and patient access.

## 5. Conclusions

Bulgarian oncology physicians reported a high level of self-reported professional readiness for the implementation of innovative oncology therapies and broadly favorable perceptions of their clinical value. However, their assessments of timely patient access were more variable. Respondents most frequently identified high budget impact, regulatory and HTA-related delays, and perceived delays in pharmaceutical-company submissions as barriers to access. Greater professional experience and higher monthly patient volume were independently associated with more favorable perceptions of the timeliness of access.

The findings suggest that, from the physicians’ perspective, improving access requires attention not only to professional readiness but also to reimbursement processes, HTA implementation, financing, and healthcare-system organization. Respondents particularly supported expanding opportunities for participation in clinical trials and improving access to specialized oncology centers. Strengthening molecular diagnostic capacity and professional information and training were also identified as relevant priorities. These findings may inform discussion among policymakers, HTA bodies, healthcare providers, and professional organizations, but they should be interpreted as clinician-reported perceptions rather than objectively established determinants of access.

Within the evolving European HTA framework, the study contributes multidisciplinary evidence regarding how physicians involved in cancer care perceive the implementation and accessibility of innovative oncology therapies in Bulgaria. Future research should combine physicians’ perspectives with objective indicators of reimbursement timelines, medicine availability, molecular-testing capacity, treatment utilization, and patient outcomes. Such evidence would provide a more comprehensive basis for evaluating access to oncology innovation and informing healthcare policy.

## Figures and Tables

**Table 1:** Demographic and professional characteristics of the study participants (*n* = 114).

Characteristic	Category	*n*	%	95% CI
Sex	Male	57	50.0	41.0–59.0
	Female	57	50.0	41.0–59.0
Age (yrs) *	Median (IQR)	45 (34–57)	-	
	25–30	13	11.6	6.9–18.9
	31–40	32	28.6	21.0–37.5
	41–55	35	31.2	23.4–40.3
	≥56	32	28.6	21.0–37.5
Professional experience (yrs) †	Median (IQR)	15 (7.5–25)	-	
	<10 years	32	28.8	21.2–37.9
	10–24 years	48	43.2	34.4–52.5
	≥25 years	31	27.9	20.4–36.9
Medical specialty ‡	Medical oncology	51	44.7	35.9–53.9
	Other oncology-related specialties	63	55.3	46.1–64.1
Practice sector	Public	69	60.5	51.4–69.0
	Private	40	35.1	26.9–44.2
	Mixed	5	4.4	1.9–9.9
Monthly patient volume	<50	29	25.4	18.3–34.1
	50–100	32	28.1	20.6–36.9
	100–200	14	12.3	7.5–19.6
	>200	39	34.2	26.1–43.3

Data are presented as frequency and percentage unless otherwise indicated. Confidence intervals for proportions are two-sided 95% Wilson score intervals. * Age data were available for 112 respondents. † Professional-experience data were available for 111 respondents. ‡ For inferential analysis, respondents selecting medical oncology, either alone or together with another specialty, were classified as medical oncologists; respondents who did not select medical oncology were classified as other oncology-related specialists.

**Table 2:** Multivariable ordinal logistic regression analysis of factors perceived as associated with physicians’ perceptions of the timeliness of access to innovative oncology therapies *.

Predictor	β	SE	Wald χ^2^	aOR	95% CI	*p*-Value
Female sex vs. male	0.482	0.374	1.66	1.62	0.78–3.37	0.197
Professional experience, per additional 10 yrs	−0.383	0.156	6.00	**0.68**	**0.50–0.93**	**0.014**
Medical oncologist vs. other specialties	−0.694	0.405	2.94	0.50	0.23–1.10	0.086
Public vs. private/mixed sector	−0.062	0.363	0.03	0.94	0.46–1.91	0.864
≥100 vs. <100 patients with cancer per month	−0.796	0.386	4.26	**0.45**	**0.21–0.96**	**0.039**

* **Model statistics:** *n* = 111; likelihood-ratio χ^2^(5) = 16.29, *p* = 0.006; log-likelihood = −143.12; AIC = 304.25; BIC = 328.63; Nagelkerke pseudo-R^2^ = 0.146. **Abbreviations:** aOR, adjusted odds ratio; CI, confidence interval; SE, standard error; AIC, Akaike Information Criterion; BIC, Bayesian Information Criterion. **Interpretation:** The outcome was coded from 1 = very good to 5 = very poor. Therefore, an aOR below 1 indicates lower odds of reporting a less favorable assessment of timeliness. The model included 111 respondents with complete data for the dependent variable and all five predictors; three respondents were excluded because professional-experience data were missing. The proportional-odds assumption was supported by the non-significant test of parallel lines (χ^2^(15) = 10.43, *p* = 0.792). Medical specialty was entered as a dichotomous variable: medical oncology, selected alone or with another specialty, versus no selection of medical oncology.

## Data Availability

The data presented in this study are not publicly available because they contain anonymized survey responses from healthcare professionals. Although direct identifiers were not collected, public release of the dataset could increase the risk of indirect participant identification due to the combination of demographic and professional characteristics. Furthermore, participants provided electronic informed consent under conditions that did not include public deposition of the dataset. A de-identified full dataset has been provided to the journal during the manuscript submission process for editorial evaluation. Additional de-identified data are available from the corresponding author upon reasonable request for academic research purposes, subject to institutional approval and applicable data protection requirements.

## Supplementary Materials

The following supporting information can be downloaded at: https://www.mdpi.com/article/10.3390/healthcare14182926/s1.

## Abbreviations

The following abbreviations are used in this manuscript:

χ^2^	Chi-square statistic
AIC	Akaike Information Criterion
aOR	Adjusted Odds Ratio
BIC	Bayesian Information Criterion
CI	Confidence Interval
EMA	European Medicines Agency
ESMO	European Society for Medical Oncology
EU	European Union
HTA	Health Technology Assessment
HTA Regulation	Regulation (EU) 2021/2282 on Health Technology Assessment
IQR	Interquartile Range
NHIF	National Health Insurance Fund
SE	Standard Error
STROBE	Strengthening the Reporting of Observational Studies in Epidemiology
WHO	World Health Organization
