# Supplementary material for: Physicians’ Perspectives on Access to Innovative Oncology Therapies: A Cross-Sectional Survey in Bulgaria
Source: Healthcare (Basel). 2026 Sep 9;14(18):2926. doi: 10.3390/healthcare14182926 (PMC13606224; doi:10.3390/healthcare14182926)
Supplement: Supplementary file 1 [file healthcare-14-02926-s001.zip › healthcare-4487562-supplementary.pdf]

Survey of oncology specialists’ views on access to innovative drug therapies

Dear respondents,

Inequalities in patients’ access to cancer therapies and ensuring timely access to treatment innovations are major challenges for healthcare systems. Although increasing numbers of oncology medicines are being developed and approved, inequalities in access persist. The number of approved cancer therapies continues to grow over time. Between 2004 and 2011, the average annual number of approvals was nearly four in both the European Union and the European Economic Area (EU/EEA), whereas 17 and 15 new therapies were approved in 2021 and 2022, respectively. In addition, approvals for extensions of indications for existing therapies have outnumbered approvals of new medicines in recent years. Oncology research suggests that this development is likely to continue in the future.

This questionnaire aims to explore your views and assessments regarding the timeliness of access, the barriers that prolong the time to access, and measures to reduce delays in access to innovative therapies for patients with cancer in Bulgaria. The questions are organised into six panels, and completing the questionnaire takes approximately 10-12 minutes.

Your responses will be analysed anonymously. The information obtained will be used in aggregated form solely for scientific publications and a doctoral dissertation entitled “Innovation in Oncology Therapies”.

The survey is being conducted by Iva Aneva, a full-time doctoral student in the Department of Social Medicine and Public Health at Medical University - Plovdiv, under the supervision of Associate Professor Ralitsa Raycheva, PhD, and Professor Rositsa Dimova, MD, PhD.

Thank you in advance for your time and assistance!

Iva Aneva, full-time doctoral student Department of Social Medicine and Public Health Medical University - Plovdiv Iva.Zdravkova-Aneva@mu-plovdiv.bg

* Indicates a required question

Informed consent to participate in a survey

PARTICIPATION

Your participation in this survey is voluntary. You may decline to participate or withdraw from the study at any time.

BENEFITS

You will not receive any direct benefits from participating in this research. However, your responses may help us learn more about access to oncology drug therapies, particularly the reasons for delays in access, barriers to access, and measures to improve access.

RISKS

The potential risks or discomforts associated with the study are minimal.

CONFIDENTIALITY

Your survey responses will be submitted via a docs.google.com/forms/ link, where the data will be stored electronically in a password-protected format. Google Forms does not collect identifying information such as your name, email address or IP address. Your responses will therefore remain anonymous. No one will be able to identify you or your responses, and no one will know whether or not you participated in the study.

CONTACT

If you have any questions about the study or its procedures, you may contact my academic supervisors at any time: Associate Professor Ralitsa Raycheva, PhD [r.raycheva@mu-plovdiv.bg], and Professor Rositsa Dimova, MD, PhD [rositsa.dimova@mu-plovdiv.bg].

1. ELECTRONIC CONSENT: By selecting “I agree”, you confirm that you are over 18 years of age, have read and understood the information provided, and voluntarily agree to participate in this study. *

Select one answer.

( ) I agree - Go to question 2.

( ) I do not agree

GENERAL INFORMATION

DEMOGRAPHIC QUESTIONS

2. What is your gender? *

Select one answer.

( ) Male

( ) Female

( ) Prefer not to answer

3. How old are you? *

____

4. How many years of professional medical experience do you have in oncology? *

____

5. What is your medical specialty? (More than one answer may be selected.) *

Select all that apply.

[ ] Medical oncology

[ ] Clinical haematology

[ ] Paediatric clinical haematology and oncology

[ ] Surgery

[ ] Nuclear medicine

[ ] Diagnostic imaging

[ ] Radiotherapy

[ ] Clinical immunology

[ ] Medical genetics

[ ] Other: ______________________________________

6. What types of cancer do you treat? (More than one answer may be selected.) *

Select all that apply.

[ ] Solid malignancies

[ ] Haematological malignancies

7. Which patients do you work with? *

Select one answer.

( ) Adults only

( ) Children only

( ) Both adults and children

8. Which sector of the healthcare system do you work in (for more than 50% of your time)? *

Select one answer.

( ) Public

( ) Private

( ) Equally in both

9. In which city is the healthcare institution where you work located?

____

10. What is the name of the healthcare institution where you work?

____

11. What proportion of your working time is devoted to clinical practice? *

Select one answer.

( ) Less than 20%

( ) 20-39%

( ) 40-59%

( ) 60-79%

( ) 80-100%

( ) Other: ____________________________________

12. How many patients with cancer do you work with per month? *

Select one answer.

( ) Fewer than 50 patients

( ) 50-100 patients

( ) 100-200 patients

( ) More than 200 patients

( ) Other: ____________________________________

13. Do you participate in any of the following activities? (More than one answer may be selected.) *

Select all that apply.

[ ] Developing national medical standards for systemic drug treatment, assessment of treatment response and follow-up of malignant diseases

[ ] Developing pharmacotherapeutic guidelines

[ ] Participating in health technology assessment (HTA) expert groups

[ ] Other: ____________________________________

INNOVATIVE DRUG THERAPIES IN ONCOLOGY

An innovative therapy is a medicine containing an active substance or a combination of active substances that has not previously undergone an authorisation procedure by the European Medicines Agency. (EMA definition - https://www.ema.europa.eu/en/glossary-terms/innovative-medicine)

14. In your opinion, what are the main advantages of innovative oncology drug therapies? (More than one answer may be selected.) *

Select all that apply.

[ ] Longer survival (overall and/or progression-free)

[ ] Fewer side effects

[ ] Improved quality of life

[ ] Possibility of using combination therapies

[ ] Possibility of personalised treatment

[ ] Other: ____________________________________

15. Do you consider yourself well informed about the innovative drug therapies available in oncology? *

Select one answer.

( ) Yes, fully

( ) Yes, to some extent

( ) No, not sufficiently

( ) No, not at all

( ) Other: ____________________________________

16. Where do you most frequently obtain information about innovative oncology drug therapies (e.g. development, clinical trials, marketing authorisations, access, real-world outcomes, etc.)? (More than one answer may be selected.) *

Select all that apply.

[ ] Specialist medical literature (publications, monographs, etc.)

[ ] Scientific conferences and seminars

[ ] Training courses and workshops

[ ] Online resources/platforms and databases

[ ] Professional and expert networks

[ ] National and international oncology organisations

[ ] Pharmaceutical companies

[ ] Other: ____________________________________

17. How would you rate the reliability of the information you receive from the different sources? (Rate each answer on a scale from 1 to 5, where 1 is “Very low” and 5 is “Very high”.) *

Select one answer in each row.

	1 Very low	2 Low	3 Moderate	4 High	5 Very high
Specialist medical literature	( )	( )	( )	( )	( )
Scientific conferences and seminars	( )	( )	( )	( )	( )
Training courses and workshops	( )	( )	( )	( )	( )
Online resources/platforms and databases	( )	( )	( )	( )	( )
Professional and expert networks	( )	( )	( )	( )	( )
National and international oncology organisations	( )	( )	( )	( )	( )
Pharmaceutical companies	( )	( )	( )	( )	( )

18. What proportion of your patients receive innovative oncology drug therapies? *

Select one answer.

( ) Less than 10%

( ) 10-30%

( ) 30-50%

( ) More than 50%

( ) Other: ____________________________________

19. What factors do you consider when selecting an innovative drug therapy for your patients with cancer? (More than one answer may be selected.) *

Select all that apply.

[ ] Therapeutic efficacy

[ ] Side effects

[ ] Availability of the medicine

[ ] Pharmacotherapeutic guidelines

[ ] Experience with the therapy in previous patients

[ ] Recommendations from colleagues or experts

[ ] Opportunities for patients to participate in clinical trials

[ ] The patient’s opinion

[ ] Other: ____________________________________

20. How would you rate the “time-to-event” outcomes achieved with innovative oncology drug therapies compared with older therapies? *

Select one answer in each row.

	Lower outcome	No difference	Higher outcome	Unable to answer
Overall survival	( )	( )	( )	( )
Progression-free survival	( )	( )	( )	( )
Event-free survival	( )	( )	( )	( )
Disease-free survival	( )	( )	( )	( )
1-year survival	( )	( )	( )	( )
5-year survival	( )	( )	( )	( )
Time to progression	( )	( )	( )	( )
Time to response to therapy	( )	( )	( )	( )
Duration of response to therapy	( )	( )	( )	( )
Time to next therapy	( )	( )	( )	( )

21. How would you rate the “response to therapy” outcomes achieved with innovative oncology drug therapies compared with older therapies? *

Select one answer in each row.

	Lower outcome	No difference	Higher outcome	Unable to answer
Objective response	( )	( )	( )	( )
Overall response rate	( )	( )	( )	( )
Pathological complete response	( )	( )	( )	( )
Disease control rate	( )	( )	( )	( )
Minimal residual disease	( )	( )	( )	( )
Recurrence rate	( )	( )	( )	( )

22. How would you rate the “patient-reported outcomes” achieved with innovative oncology drug therapies compared with older therapies? *

Select one answer in each row.

	Less frequently	No difference	More frequently	Unable to answer
Symptom reduction	( )	( )	( )	( )
Improved quality of life	( )	( )	( )	( )
Improved functioning	( )	( )	( )	( )
Reduction in side effects	( )	( )	( )	( )

23. How would you assess the costs of innovative oncology drug therapies in relation to their effectiveness? *

Select one answer.

( ) Very good cost-effectiveness

( ) Good cost-effectiveness

( ) Satisfactory cost-effectiveness

( ) Poor cost-effectiveness

( ) Very poor cost-effectiveness

( ) Unable to judge

24. How often do you need to adapt the treatment plan because of patients' specific needs or reactions to innovative oncology drug therapies? *

Select one answer.

( ) Fewer than 1% of patients

( ) 1% to 5% of patients

( ) 5% to 10% of patients

( ) More than 10% of patients

( ) Other: ____________________________________

25. What benefits do you see for patients from using innovative oncology drug therapies? (More than one answer may be selected.) *

Select all that apply.

[ ] Longer overall survival

[ ] Longer progression-free survival

[ ] Use of less burdensome protocols for administering therapies

[ ] Faster recovery

[ ] Reduced risk of side effects

[ ] Other: ____________________________________

ACCESS TO INNOVATIVE ONCOLOGY DRUG THERAPIES

Access to drug therapy is provided through the European Medicines Agency's centralised marketing authorisation procedure. The procedure authorises the pharmaceutical company holding the marketing authorisation to market the medicine and make it available throughout the EU on the basis of a single marketing authorisation. Patients in Bulgaria can access an innovative oncology therapy after it has been included in the Positive Drug List. This procedure is set out in the Medicinal Products in Human Medicine Act and the Ordinance on the Terms, Rules and Procedure for Regulating and Registering the Prices of Medicinal Products.

26. How would you rate the timeliness of the introduction of innovative oncology drug therapies in Bulgaria? *

Select one answer.

( ) Very good

( ) Good

( ) Satisfactory

( ) Poor

( ) Very poor

27. How would you rate Bulgarian patients' access to innovative oncology drug therapies compared with other European countries? *

Select one answer.

( ) Much faster

( ) Faster

( ) Comparable

( ) Slower

( ) Much slower

28. In your practice, has a patient ever chosen treatment abroad because of a lack of access to an innovative oncology drug therapy in Bulgaria? *

Select one answer.

( ) Yes

( ) No

( ) Unable to answer

29. If your answer to the previous question was “Yes”, please estimate how often this occurs: *

Select one answer.

( ) Fewer than 5% of patients

( ) 5% to 10% of patients

( ) 10% to 20% of patients

( ) More than 20% of patients

( ) Other: ____________________________________

30. Please list oncology drug therapies that you believe Bulgarian patients should have access to and that are available in other European countries but are not yet included in Bulgaria’s Positive Drug List.

____

BARRIERS TO ACCESSING INNOVATIVE ONCOLOGY DRUG THERAPIES

31. Which of the following barriers most frequently hinder your patients’ access to innovative oncology drug therapies? (More than one answer may be selected.) *

Select all that apply.

[ ] Delays by pharmaceutical companies in submitting applications for the inclusion of an innovative oncology therapy in the Positive Drug List

[ ] Regulatory restrictions and delays in the health technology assessment (HTA) procedure and the inclusion of the therapy in the Positive Drug List

[ ] High budget impact on the National Health Insurance Fund (NHIF)

[ ] Insufficient information and training for medical staff

[ ] Lack of awareness and understanding among patients

[ ] Other: ____________________________________

32. How would you rate the importance of the listed barriers in restricting access to innovative oncology drug therapies? (Rate each answer on a scale from 1 to 5, where 1 is "Very low importance" and 5 is "Very high importance".) *

Select one answer in each row.

	1 Very low importance	2 Low importance	3 Moderate importance	4 High importance	5 Very high importance
Delays by pharmaceutical companies in submitting applications for the inclusion of an innovative oncology therapy in the Positive Drug List	( )	( )	( )	( )	( )
Regulatory restrictions and delays in the health technology assessment (HTA) procedure and the inclusion of the therapy in the Positive Drug List and pharmacotherapeutic guidelines	( )	( )	( )	( )	( )
High budget impact on the National Health Insurance Fund (NHIF)	( )	( )	( )	( )	( )
Insufficient information and training for medical staff	( )	( )	( )	( )	( )
Lack of awareness and understanding among patients	( )	( )	( )	( )	( )

33. For what proportion of your patients is access to innovative oncology drug therapies hindered by any of the barriers listed in the previous question? *

Select one answer.

( ) None

( ) Fewer than 5% of patients

( ) 5% to 10% of patients

( ) 10% to 20% of patients

( ) More than 20% of patients

( ) Other: ____________________________________

34. How would you rate the importance of measures that could improve access to innovative oncology drug therapies? (Rate each answer on a scale from 1 to 5, where 1 is "Very low importance" and 5 is "Very high importance".) *

Select one answer in each row.

	1 Very low importance	2 Low importance	3 Moderate importance	4 High importance	5 Very high importance
More clinical trials at the local level	( )	( )	( )	( )	( )
Improved access to specialised oncology centres	( )	( )	( )	( )	( )

35. Do you think a dedicated fund is needed to reimburse only innovative oncology drug therapies, providing faster access to them? *

Select one answer.

( ) Yes, such a fund is absolutely necessary and would significantly facilitate patients' access to new therapies

( ) Yes, but clear reimbursement criteria and mechanisms should be considered

( ) No, the current mechanisms are sufficient, but they need to be optimised

( ) No, such a fund would create additional administrative and financial difficulties

( ) Unable to judge

( ) Other: ____________________________________

PHARMACOTHERAPEUTIC GUIDELINES

36. From which source do you most often learn that an innovative oncology drug therapy is available and accessible to patients? *

Select one answer.

( ) National pharmacotherapeutic guidelines

( ) International guidelines (e.g. ESMO, ASCO, NCCN)

( ) Institutional protocols and standards

( ) Personalised protocols based on experience and data

( ) Other: ____________________________________

37. To what extent do you follow the updated recommendations included in pharmacotherapeutic guidelines when using innovative oncology drug therapies? *

Select one answer.

( ) Fully (I follow and apply all new recommendations)

( ) Partially (I follow updates, but apply them on a case-by-case basis)

( ) Rarely (I mainly use older recommendations or personal experience)

( ) Never (I do not follow the new recommendations)

( ) Other: ____________________________________

38. In your opinion, are the pharmacotherapeutic guidelines you use (e.g. the Pharmacotherapeutic Guidelines for Medical Oncology, Paediatric Clinical Haematology and Oncology, and Haematology) updated frequently enough to improve patients' access to innovative oncology drug therapies? *

Select one answer.

( ) Yes, the pharmacotherapeutic guidelines reflect changes to the Positive Drug List quickly enough, and patients gain timely access to innovative oncology therapies

( ) Yes, the pharmacotherapeutic guidelines reflect changes to the Positive Drug List, but the time taken means that patients cannot benefit promptly from the available innovative therapy

( ) No, the pharmacotherapeutic guidelines do not reflect changes to the Positive Drug List in a timely manner, and patients have to wait for an update before accessing the innovative therapy

( ) No, the lack of a mechanism for rapidly updating the pharmacotherapeutic guidelines is a barrier to patients' access to innovative oncology therapies

( ) Other: ____________________________________

39. What difficulties do you encounter when applying pharmacotherapeutic guidelines after they have been updated to include innovative oncology drug therapies? (More than one answer may be selected.) *

Select all that apply.

[ ] Lack of clear or specific instructions

[ ] Need for additional training

[ ] Problems with the availability of the necessary resources

[ ] Conflicts between different guidelines

[ ] I encounter no difficulties; everything is set out very precisely and clearly

[ ] Other: ____________________________________

40. How would you rate the importance of each of the listed potential changes to pharmacotherapeutic guidelines for improving patients' access to innovative oncology drug therapies? (Rate each answer on a scale from 1 to 5, where 1 is "Very low importance" and 5 is "Very high importance".) *

Select one answer in each row.

	1 Very low importance	2 Low importance	3 Moderate importance	4 High importance	5 Very high importance
More frequent updates	( )	( )	( )	( )	( )
More detailed recommendations and alignment with the format of guidelines such as ESMO, ASCO and NCCN	( )	( )	( )	( )	( )
Better integration of data from new clinical trials	( )	( )	( )	( )	( )
Greater flexibility and personalisation of recommendations	( )	( )	( )	( )	( )

CLINICAL TRIALS

41. If a patient is interested in participating in clinical trials or would be a suitable candidate, do you actively help them find a trial? *

Select one answer.

( ) Yes

( ) No

( ) Unable to answer

( ) Other: ____________________________________

42. Do you have experience as an investigator in clinical trials of oncology drug therapies? *

Select one answer.

( ) Yes, I actively participate in clinical trials

( ) Yes, I have experience, but I am not currently actively involved

( ) No, I have no experience in clinical trials

43. During your professional career, how many clinical trials have you participated in as an investigator? *

Select one answer.

( ) None

( ) 1-5

( ) 6-10

( ) 11-20

( ) More than 20

( ) Other: ____________________________________

44. What are your main motivations for participating in clinical trials as an investigator? (More than one answer may be selected.) *

Select all that apply.

[ ] Access to new and innovative therapies for patients

[ ] Scientific interest and professional development

[ ] Financial remuneration

[ ] Expanding my network of professional contacts

[ ] Other: ____________________________________

45. Do you have the opportunity to enrol your patients in clinical trials of oncology drug therapies? *

Select one answer.

( ) Yes, I have enrolled up to 5 patients over my entire career

( ) Yes, I have enrolled 5 to 15 patients over my entire career

( ) Yes, I have enrolled 15 to 30 patients over my entire career

( ) Yes, I have enrolled more than 30 patients over my entire career

( ) No, I have never enrolled patients in clinical trials

( ) Other: ___________________________________

46. What are the main barriers to your patients' participation in clinical trials? (More than one answer may be selected.) *

Select all that apply.

[ ] Lack of suitable trials in the region

[ ] Regulatory restrictions

[ ] Insufficient support from the hospital/healthcare institution

[ ] Insufficient patient awareness

[ ] Other: ___________________________________

JOINT CLINICAL ASSESSMENT (JCA)

Joint Clinical Assessment (JCA) is a European Union process for assessing the clinical effectiveness and safety of new health technologies, including drug therapies. It aims to provide scientifically sound information to support decisions on the use of these technologies in Member States' health systems, avoid duplication of effort and resources, and ensure faster patient access to innovative treatments. Regulation (EU) 2021/2282, effective from 12 January 2025, governs cooperation on JCA. The responsible authority in Bulgaria is the National Council on Prices and Reimbursement of Medicinal Products (NCPR).

47. Are you familiar with the Joint Clinical Assessment (JCA) procedure? *

Select one answer.

( ) Yes

( ) No

48. How would you rate the likelihood of the following changes occurring as a result of implementing Joint Clinical Assessment? (Rate each answer on a scale from 1 to 5, where 1 is “Very low likelihood” and 5 is “Very high likelihood”.) *

Select one answer in each row.

	1 Very low likelihood	2 Low likelihood	3 Moderate likelihood	4 High likelihood	5 Very high likelihood
Faster/improved patient access to innovative oncology therapies	( )	( )	( )	( )	( )
Greater predictability and transparency in the assessment of new therapies	( )	( )	( )	( )	( )
Reduced administrative burden in accessing new therapies	( )	( )	( )	( )	( )
Little or no impact on access, as national authorities will continue to determine pricing and reimbursement	( )	( )	( )	( )	( )
No significant impact on access to innovative therapies	( )	( )	( )	( )	( )
